# Early bacterial communities in the lower airways and intestines of caesarean section neonates with respiratory disease

**DOI:** 10.3389/fcimb.2025.1562791

**Published:** 2025-10-20

**Authors:** Yonghua Wei, Yang Wang, Shiyi Sun, Zhimin Zhao, Weiqun Lu, Yan Ma, Jie Chen, Li Xu, Kerong Li

**Affiliations:** ^1^ Department of Longen Technology Group Co., Kunming, China; ^2^ Department of Pediatric, Yan’an Affiliated Hospital of Kunming Medical University, Kunming, China

**Keywords:** acute lower respiratory tract infection, neonates, airway secretion, feces, bacterial community

## Abstract

**Introduction:**

Acute lower respiratory tract infection (ALRTI) are one of the most severe diseases affecting neonates worldwide. Many bacterial pathogens that cause respiratory infections in neonates are common residents in the respiratory tract. Therefore, the aim is to determine the early bacterial community in the lower airways and intestines of neonates.

**Methods:**

Airway secretions, oral swabs, and fecal samples were collected from 19 neonates with acute respiratory tract infection and 2 control subjects. All three types of samples were amplified and sequenced using specific primer sets targeting the 16S rRNA gene.

**Results:**

Bacterial composition of the feces and airway secretions in the diseased neonates significantly differed from that of the neonates in the control group. The feces microbiota in the diseased neonates had accumulated *Alcaligenaceae*_ge and *Enterococcus*. The airway secretion microbiota of control and diseased neonates was mainly *Alcaligenaceae*_ge and *Streptococcus*, and *Alcaligenaceae*_ge was the most abundant genus in diseased neonates. In addition, the bacterial composition of the twins’ neonates samples was more similar than that of other neonates.

**Discussion:**

Insight into the source and stability of microbiota in the neonatal period might elucidate health or susceptibility for developing a respiratory disease. Microbiota analysis also promises to complement the present means of detecting respiratory pathogens.

## 1 Introduction

Despite advances in modern medicine, acute respiratory tract illnesses (ARIs) continue to be a global health concern. The human respiratory tract includes the upper and lower respiratory tracts. Lower respiratory infections poses a significant threat to human health due to high morbidity and mortality rates. According to the 2019 statistical report of the World Health Organization (WHO), lower respiratory infections (ranked second) were among the top ten causes of burden in children (Kisa et al., 2020).The microbiological landscape of neonatal ALRTI is complex, with various microorganisms implicated in its etiology. Notably, *Klebsiella* ALRTI and coagulase-negative *Staphylococcus* (CoNS) are among the most frequently isolated pathogens in cases of neonatal sepsis and ALRTI (Brito et al., 2010; Karimi et al., 2023). These organisms can be transmitted vertically from the mother or acquired postnatally from the environment, including healthcare settings, where they may colonize surfaces and medical equipment (Kurma et al., 2019; Kamgaing et al., 2021). The presence of these bacteria in fecal matter and saliva highlights the importance of hygiene and infection control measures in neonatal intensive care units (NICUs) to prevent outbreaks of nosocomial infections (Kamgaing et al., 2021; Agrawal et al., 2022).

Fecal microorganisms, particularly those from the family Enterobacteriaceae, including *Escherichia coli* and *Klebsiella* ALRTI, are significant contributors to neonatal infections. Studies have shown that these bacteria can be present in the gut microbiome of neonates and may translocate into the bloodstream, leading to sepsis and ALRTI (Amandito et al., 2022). The gut microbiome’s composition can be influenced by various factors, including antibiotic use, which may disrupt normal flora and promote the growth of pathogenic organisms (Parm et al., 2010). Furthermore, the role of saliva as a potential vector for pathogens in neonates cannot be overlooked, as oral secretions can harbor bacteria that may contribute to respiratory infections (Hooven & Polin, 2017).

The clinical implications of these findings are profound. The identification of specific pathogens in neonatal ALRTI can guide appropriate antibiotic therapy and improve outcomes. For instance, the prevalence of multidrug-resistant strains of *Klebsiella* ALRTI necessitates careful selection of empirical antibiotic regimens to combat these infections effectively (Khaertynov et al., 2017). Moreover, understanding the microbiological profile of neonates with ALRTI can aid in developing targeted prevention strategies, such as enhanced hygiene practices and the judicious use of antibiotics to minimize the risk of colonization by harmful microorganisms (Parm et al., 2010; Agrawal et al., 2022).

In summary, neonatal ALRTI is influenced by a variety of microorganisms, including those from fecal and saliva sources. The interplay between these pathogens and the neonate’s immature immune system underscores the need for vigilant infection control practices in NICUs to mitigate the risks associated with these infections.

## 2 Materials and methods

### 2.1 Subject recruitment and clinical information

Neonates with lower respiratory tract infections were recruited in the neonatal department of Yan’an Hospital affiliated to Kunming Medical University in Yunnan Province. This study was approved by Ethics committee of Yan’an Hospital affiliated to Kunming Medical University in Yunnan Province(2019-056-01), and the informed consent forms were signed by the parents of patients. Cases were included based on the inclusion criteria proposed by (Shao et al., 2019), with slight modifications, as follows: i) Clinical Manifestations: Patients present with tachypnea, groaning, dyspnea, apnea, unstable body temperature, jaundice, poor responsiveness, and coarse breath sounds, diminished breath sounds, or rales in the lungs. ii) X - ray Findings: Predominantly interstitial pneumonia; Small patchy or linear vague shadows in both lungs; Thickened bronchial walls, granular shadows accompanied by air bronchograms and emphysema. iii) Suspected Infection: Maternal premature rupture of membranes for more than 24 hours, chorioamnionitis, maternal history of infection in the third trimester of pregnancy, fetal intrauterine distress, neonatal asphyxia, neonatal meconium aspiration, etc. Cases that meet all the above manifestations i), ii) and iii) and have negative results of routine microbial tests were included. The exclusion criteria are as follows: i) Patients with positive results of routine microbiological tests; ii) Patients who have not signed the informed consent form; iii) Insufficient collected test samples; iv) Lack of clinical information. Inclusion Criteria for the Control Group: i) The infants’ mothers had no history of premature rupture of membranes, chorioamnionitis, or infection in the third trimester of pregnancy; and the infants had no history of intrauterine distress, neonatal asphyxia, neonatal meconium aspiration, etc. ii) The infants were in good general condition, with normal feeding and good responsiveness, free from pathological jaundice, with normal growth and development, and no abnormal signs in the lungs.

### 2.2 Sample collection

Two types of samples were collected from each neonate. The fecal samples and tracheal aspirates samples of neonates with ALRTI in the Pediatric department were collected respectively. Hospital staff collected the feces of the neonates with sterile cotton swabs from the diapers. The airway secretion of the neonates were obtained using bronchoscopy and placed in a sterile sample collection container. They were stored at low temperature and then the fecal and airway secretion samples were transported on ice to the laboratory and stored at -80°C until genomic DNA was extracted.

### 2.3 DNA preparation, PCR amplification, and sequencing

The total DNA of the fecal, oral swab and airway secretion samples was extracted. For the fecal samples, the QIAamp PowerFecal DNA Kit was used to extract the genomic DNA of the fecal samples. The extraction operation steps were referred to the instruction manual. After extraction, the DNA was quantified with a Nanodrop ND - 1000 spectrophotometer, and the extracted DNA was stored in a -20°C refrigerator for later use. The total DNA extraction method for the airway secretion samples was as described previously (Schmidt et al., 1991), with appropriate modifications according to the experiment. Total DNA extraction from airway secretion samples was performed using the CTAB method (Schmidt et al., 1991) with slight modifications. DNA was extracted from 1.0 g of sample in a 1.5 mL centrifuge tube. The specific steps are as follows: the sample was resuspended in 450 µL of lysis buffer (0.1 mol/L Tris/HCl, 0.1 mol/L EDTA, and 0.75 mol/L sucrose), treated with lysozyme (50 mg/mL), and incubated in a 37 °C water bath for 30 minutes. Subsequently, the sample was treated with 1% SDS and 1% CTAB, followed by sequential treatment with phenol: chloroform: isoamyl alcohol (25:24:1, volume ratio) and chloroform: isoamyl alcohol (24:1, volume ratio) to remove impurities. After treatment with ethanol, the sample was resuspended in 50 µL of TE buffer (0.01 mol/L Tris-HCl, pH 8.0; 0.001 mol/L EDTA, pH 8.0). DNA yield was assessed using Nanodrop 2000 (Thermo Fisher, United States). Primers 515F (Reysenbach et al., 1992) and 909R (Brunk and Eis, 1998) were used for PCR amplification. All primers used containing Illumina adapters sequences and dual-index barcodes to distinguish each sample. For each PCR reaction, 15 ng DNA was added as template. The PCR reaction conditions were as follows: predenaturation at 95°C for 15 s, followed by 30 cycles of 95°C for 3 min, annealing at 51°C for 30 s, extension at 72°C for 30 s, then a final extension step at 72°C for 5 min. During the DNA extraction and PCR batch for each sample, the same reagents and consumables were used, and the PCR amplification procedure was the same for all DNA, including negative controls. No PCR bands were detected in all negative control samples. The amplicon were purified with UltraClean PCR Cleanup Kit (MOBIO, United States), and the equivalent amount of PCR products were sequenced in the Illumina Miseq™ system (Illumina, United States).

### 2.4 Sequence analysis and statistics

All sequences were demultiplexed with the barcode of each sample. Sequence processing was carried out according to the characteristics of the MiSeqSOP combined with the Mothurv1.42.0 software. The SSUrRNA database sequences and classification information of SILVA (v132) were directly downloaded from the Mothur website. After sequence alignment, chimera checking was performed. Similar sequences were clustered into OTUs (operational taxonomic units) with a minimum identity of 97% or 100%. Measurements of α-diversity (within sample diversity) such as observed, Shannon, Simpson ACE, Chao1 indexes and OTU numbers were calculated at OTU level using the Mothur. After removing duplicate sequences and low-quality or short-read-length (<400bp) sequences, a total of 140,221 high-quality sequences were obtained. The phylogenetic tree was constructed by the neighbor-joining method with the MEGA6.0 software. The thetaYC method was used to analyze the distance matrix, and the principal component analysis (PCA) was performed by selecting the larger principal component. The shared OTU index was calculated with sorabund, and the abundance-based Sorenson dissimilarity index was returned. A singleton OTU is an OTU with only one sequence, and a doubleton OTU is an OTU with two sequences. After getting the generated OTU table, we used the PICRUSt online server (Langille et al., 2013) (http://galaxy.biobakery.org/) to predict the functional gene composition of the metagenomics. The STAMP software was employed to identify the pathways with statistically significant differences between groups (Parks et al., 2014). The Welch t-test was used to compare the two groups of fecal and tracheal aspirates samples, with a Storey FDR < 0.1 as the significance cutoff value. Bray-Curtis dissimilarity-based Principal Coordinates Analysis (PCoA) was performed to visualize beta-diversity patterns between the preterm group (n = 13) and the non-preterm group (n = 6) (Gao et al., 2024). The community composition differences between different sample groups were tested with the similarity analysis (ANOSIM) function in the Mothurv1.42.0 program. The population levels between different sample groups were analyzed with LEfSe. The community types were defined according to the Dirichlet multinomial mixture model, as described by (Holmes et al., 2012). This method was adopted because it allows clustering from unevenly sampled populations.

## 3 Results

### 3.1 Description of study population

A total of 19 samples from neonates with ALRTI and 2 samples from neonates with jaundice were collected at the Neonatal Department of Yan’an Hospital, Kunming Medical University, Kunming. Specifically, fecal samples were obtained from patients numbered 1 to 13, while tracheal aspiration samples were collected from patients numbered 1 to 21. Detailed information about these neonates is presented in **Supplementary Table 1**. Notably, neonates 13 and 14, who solely exhibited jaundice without ALRTI and did not receive antibiotic treatment, were designated as control cases. Given their respiratory health status, oral samples were collected instead of airway secretion samples for these two patients. The 21 participants were included in the study **Table 1**. All patients provided stool and airway secretion samples, while the control group only provided stool and oral samples. Participants were neonates, so the mean age of participants was 0.76 days (SD ± 2.47), and 53% (11) were male. The birth weight of control group was significantly higher than that of respiratory infection group (P <0.05); NEU in control group was significantly higher than that in respiratory tract infection group (P <0.05). In addition, the TBil level of the control group was more significant than that of the respiratory infection group. Overall, these data suggest that the clinical characteristics of patients with respiratory infections are significantly different from those of the control group.

**Table 1:** Clinical characteristics.

Characteristic	Patients	Control	P value
Age(days)	0.58 ± 2.52	2.5 ± 0.70	0.3
Female(%)	47%	100%	—
Birth weight (g)	2218.42 ± 665.53	3385 ± 685.89	<0.05
Gestation days	250.16 ± 16.10	282 ± 1.41	<0.05
Physical examination findings			
Total hospital days	14.05 ± 8.64	6 ± 2.83	0.2
White blood count(10^9^/L)	11.30 ± 4.23	9.93 ± 4.2	0.6
Ratio of neutrophils	38.77 ± 9.11	53.7 ± 14.42	<0.05
Total protein (U/L)	49.4 ± 7.04	55.25 ± 1.77	0.2
Total bilirubin	82.33 ± 44.04	290.1 ± 34.5	<0.0001
Lactate dehydrogenase(U/L)	1089.59 ± 525.58	1086 ± 176.78	0.9

### 3.2 Sequence information and microbial alpha diversity

**Figure 1:**
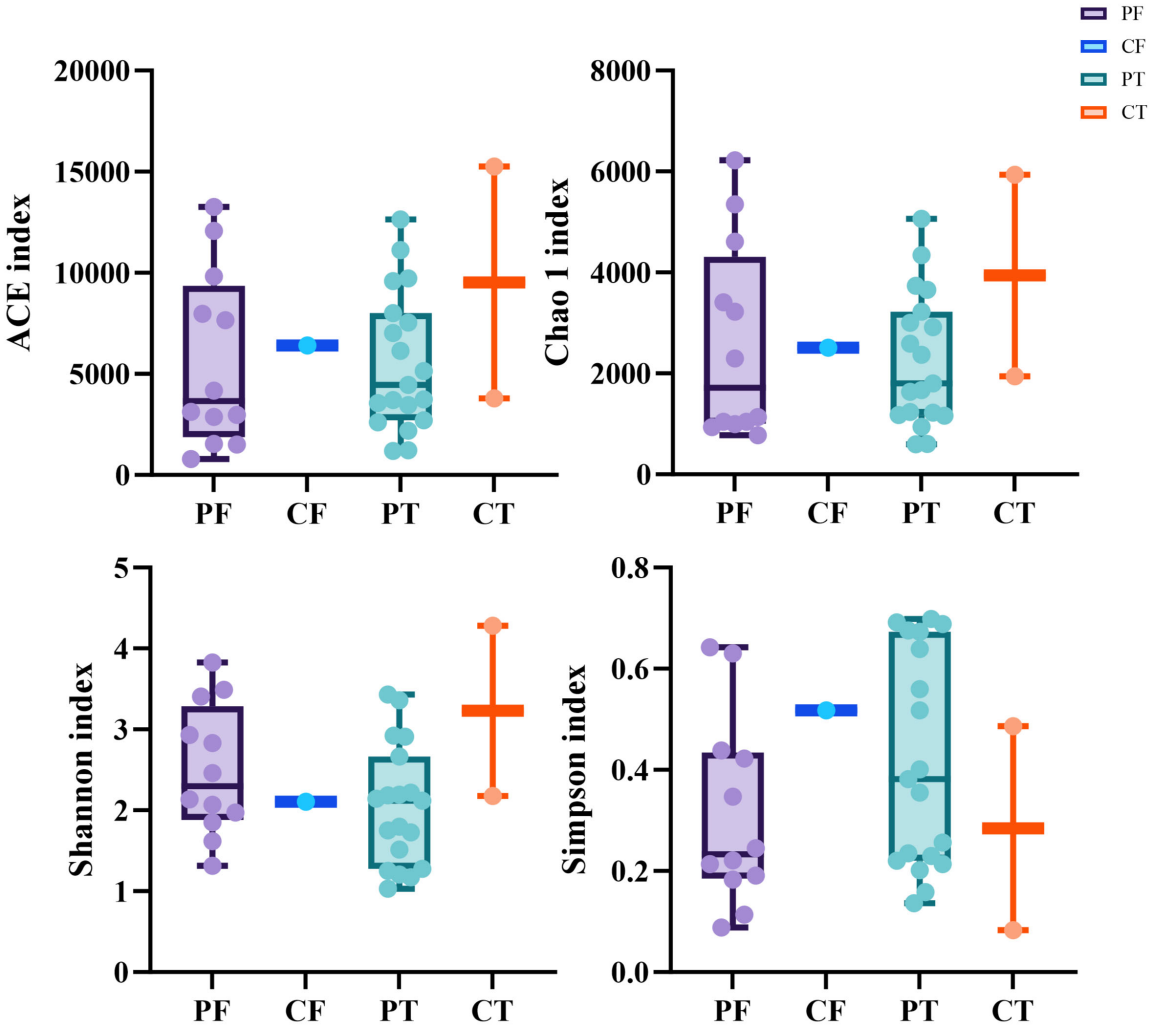
Alpha diversity between different samples based on ACE, Chao1,Shannon, Simpson indices **(A–D)**. t-test, p values represented as *p < 0.05, **p < 0.01, ***p < 0.001. PF, Feces from patients with ALRTI; PT, Airway secretion from patients with ALRTI; CF, Feces of neonates in the control group; CT, Oral swab of neonates in the control group.

The diversity indices such as the number of OTUs, Chao1, ACE, Shannon and Simpson were determined. No significant difference was found in the average indices between the neonates with ALRTI and the neonates in the control group(**Figure 1**), which may be due to the small number of control samples and the unrepresentative sample data.

**Figure 2:**
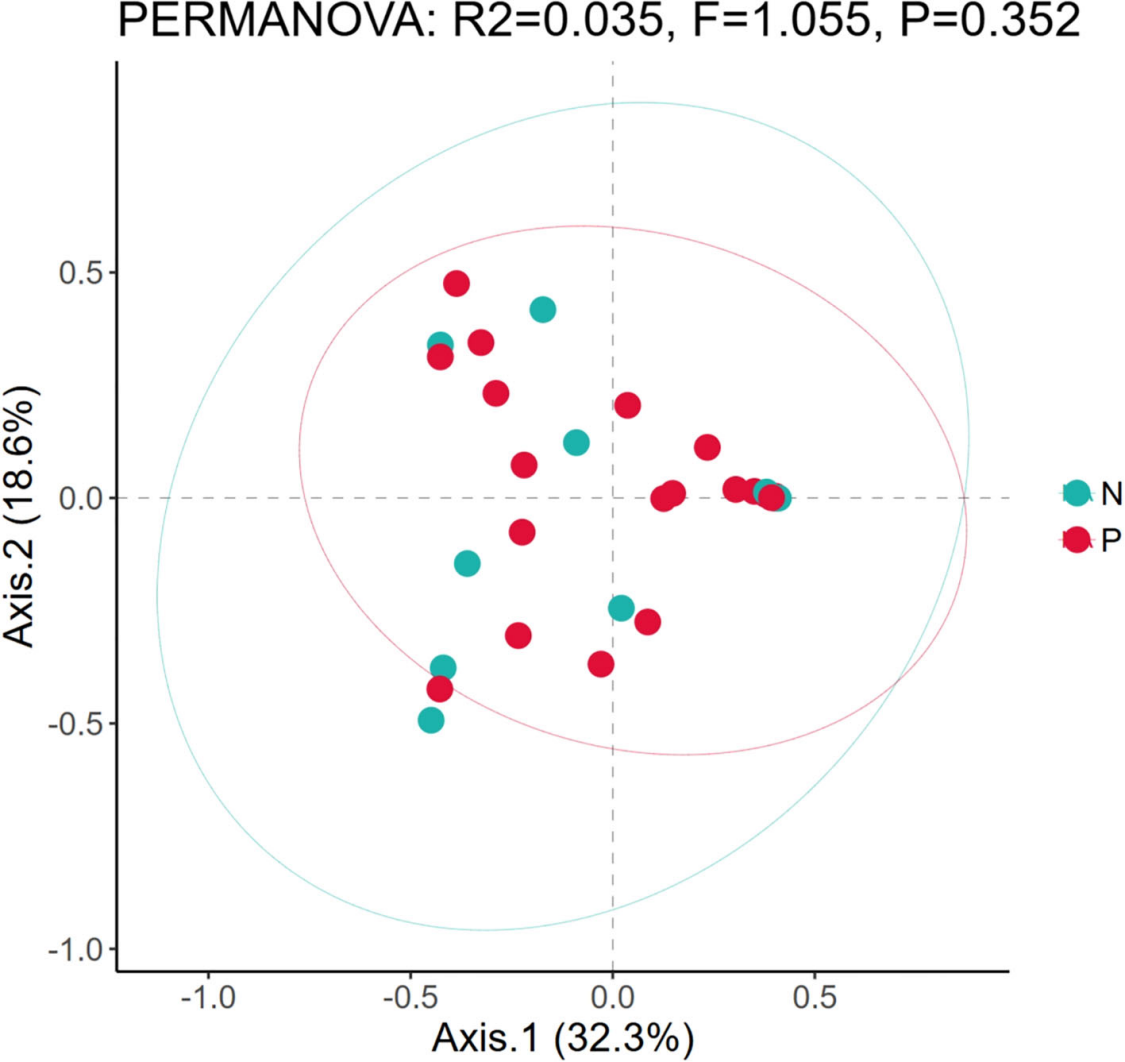
Comparison of microbial diversity between preterm neonates and non-preterm neonates. Based on the principal component analysis diagrams of the relative bacterial abundance in airway secretions and feces samples from different groups; P, The samples of premature; N, The samples of non-premature.

In addition, since there are samples of premature neonates among the neonatal samples in this study, and considering the impact of prematurity on the structure of microbial communities, we conducted β-diversity analysis on the samples of premature (P) and non-premature (N) neonates. The PCoA plot (**Figure 2**) revealed a slight separation between Group N (green) and Group P (red) along Axis.1 (32.3% variance explained). Group N clustered in the left quadrant (negative Axis.1), while Group P dominated the right quadrant (positive Axis.1). However, significant overlap was observed between groups. PERMANOVA confirmed no statistically significant difference in community composition (R²=0.035, F = 1.055, P = 0.352). The first two axes cumulatively explained 50.9% of total variance.

### 3.3 Taxonomic analysis and comparison of the fecal and airway secretions samples of the sick and control neonates

**Figure 3:**
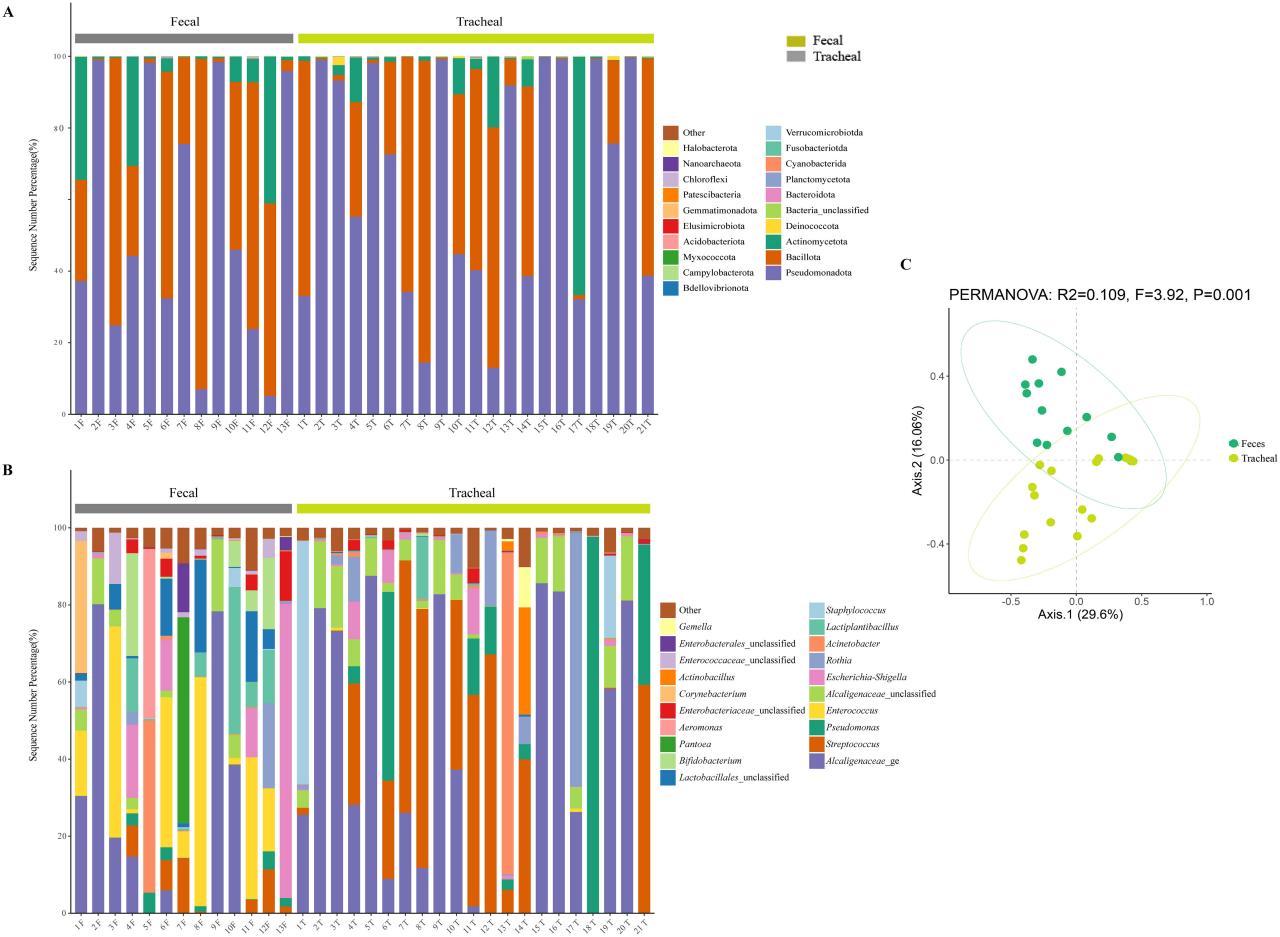
Microbial composition and diversity analysis of the respiratory tract and the feces **(A, B)**, and PCA plots based on relative taxa abundance of fecal and tracheal aspirates samples **(C)**. The composition ratio of each microorganism at phylum and genus level: Tracheal, Tracheal aspirates samples; Fecal, Fecal samples.

All samples were compared with the SSUrRNA sequence database of Silva. **Figure 2** shows the distribution of bacteria in different taxonomic levels in ALRTI patients and control neonates in different groups. At the phylum level (**Figure 3A**), in all fecal samples, three main phyla were detected, Pseudomonadota, Bacillota and Actinomycetota, accounting for more than 99% of all fecal sample sequences. Among them, Pseudomonadota was the most abundant in the fecal samples of patients 2, 5, 7, 9 and 13, with a proportion of 75% - 98%. The dominant phylum of patients 3, 6, 8 and 11 was Bacillota, with a proportion of 63% - 92%. Actinomycetota also had a significant proportion in the neonatal fecal samples, especially in patients 1, 4 and 12, with a proportion of 30% - 41%. In all airway secretion samples, the Pseudomonadota phylum was the most abundant in all samples, followed by Bacillota and Actinomycetota. Among them, Pseudomonadota was the most abundant in the airway secretion samples of patients 2, 5, 9, 13, 15, 16, 18 and 20, accounting for 91% - 99%. The dominant phylum of patients 7, 8, 12 and 21 was Bacillota, with a proportion of 60% - 84%.

At the genus level (**Figure 3B**), 20 main bacterial genera were detected from 13 fecal samples and 21 airway secretion samples, and the distribution of bacterial genera differed greatly among patients. In all fecal samples, the fecal samples of the sick neonates showed that the main genera were *Alcaligenaceae_*ge, *Enterococcus*, *Escherichia-Shigella*, *Lactiplantibacillus* and *Lactobacillales*_unclassified. Among them, the main bacterial genus of patient 13 was *Escherichia - Shigella*, accounting for about 76%. In all tracheal aspirates samples, the tracheal aspirates samples of the sick neonates showed that the main genera were *Alcaligenaceae_*ge, *Streptococcus*, *Pseudomonas*, *Alcaligenaceae*_unclassified and *Rothia*. Among them, the main bacterial genera of patients 13 and 14 were *Acinetobacter* and *Streptococcus* respectively, accounting for 83% and 40%.

To further compare the bacterial community diversity of the neonatal fecal and airway secretion samples, we performed principal coordinate analysis on the weighted UniFrac β diversity matrix (**Figure 3C**). The principal coordinates explained 29.6% and 16.06% of the variation respectively, which could better explain the distribution of the bacterial composition. Based on the PC1 and PC2 analysis, we found that there was a significant difference between the PF and PT groups (P < 0.05). This result indicates that the bacterial compositions of the airway secretion and fecal samples are different.

### 3.4 Distribution of OTUs in the fecal and airway secretion samples of the sick and control neonates

**Figure 4:**
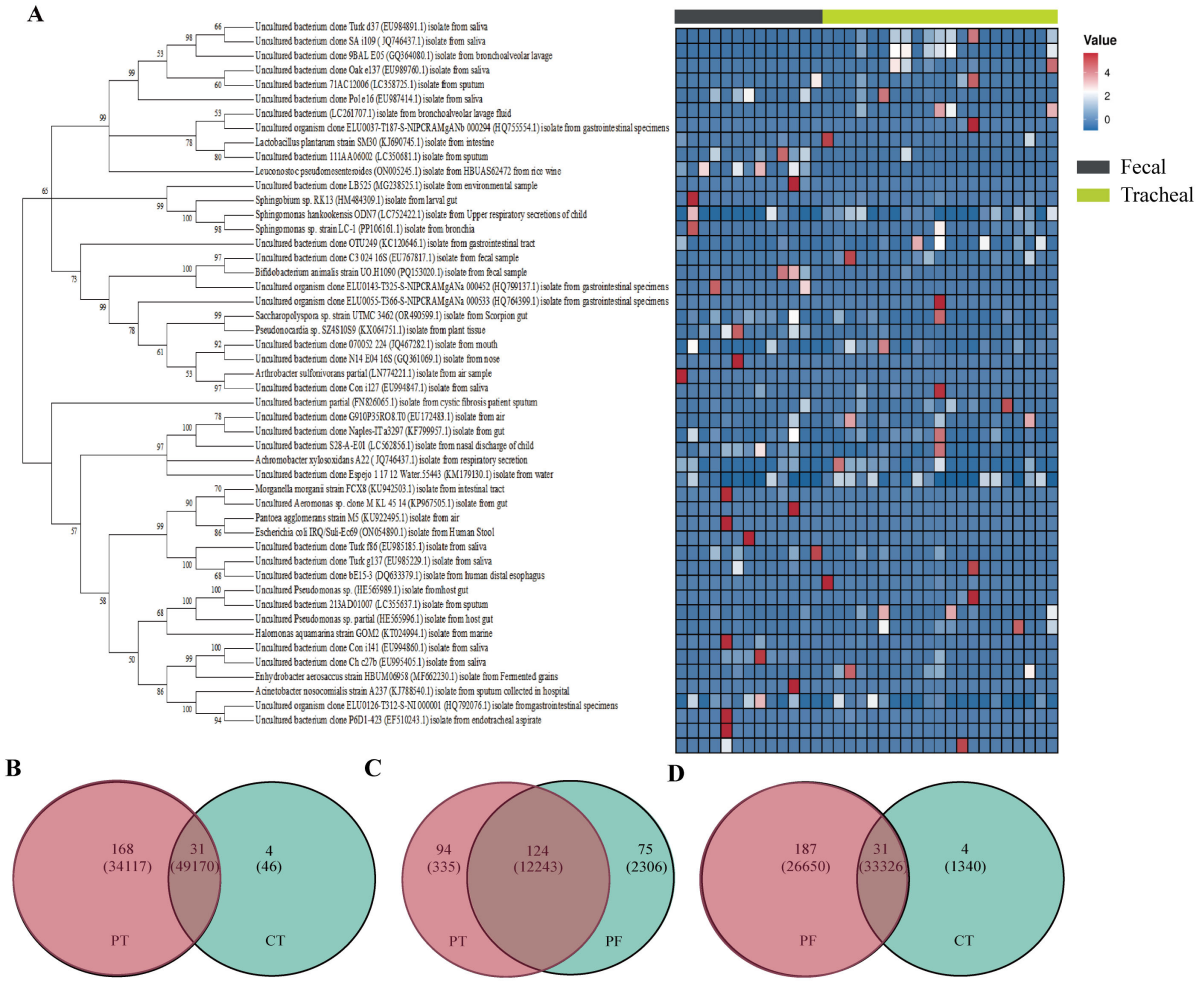
Phylogenetic tree of 16S rRNA gene sequences based on a cut-off value of 0.03. A total of 16 OTUs were used to construct a phylogenetic tree and heatmap **(A)**. Venn diagram **(B–D)** analysis at a distance of 0.03 represented OTUs and sequences among each of the two groups of samples. The OTUs and sequences of the PF and CT, PT and CT, PT and PF groups are shown. Overlap represented the shared OTUs and sequences in the different groups. PF, Feces from patients with ALRTI; PT, Airway secretion from patients with ALRTI; CF, Feces of neonates in the control group; CT, Oral swab of neonates in the control group.

To further gain an in-depth understanding of the community composition of neonatal fecal and airway secretion samples, taxonomic classification of OTUs was performed, followed by a comparison of the average abundance of bacterial communities in each sample. A phylogenetic tree was constructed using representative OTUs of each sample for overall comparison. The detailed information and specific distribution of the top 50 OTUs in all samples are as shown(**Figure 4**). There are differences in the distribution of major OTUs between neonatal fecal and airway secretion samples. Seven of the top ten OTUs were identified as uncultured sequences derived from sputum or saliva. *Escherichia coli* was the most abundant in the control group, but significantly decreased in the ALRTI group. Uncultured bacterium isolate from saliva was mainly distributed in airway secretion samples, while *Lactobacillus plantarum* was more abundant in fecal samples.

### 3.5 Prediction of bacterial functional status in tracheal aspirates samples and fecal samples after respiratory tract infection

**Figure 5:**
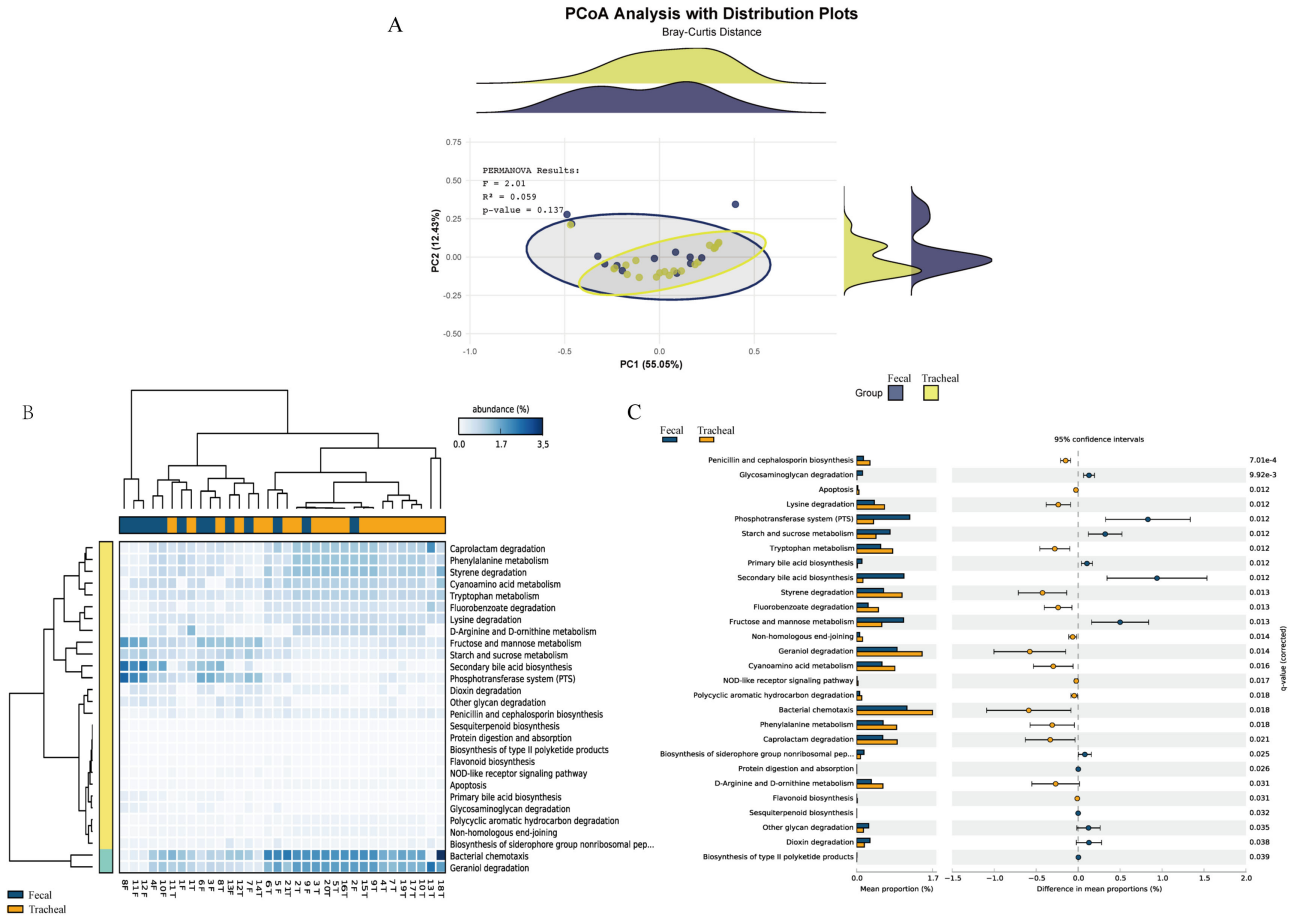
Respiratory tract infections lead to changes in the microbial functions of different sites. **(A)** Principal coordinates analysis of the prediction of bacterial functions in tracheal aspirates and fecal samples; **(B)** Heatmap of the prediction of bacterial functions in tracheal aspirates and fecal samples; **(C)** Comparison of metagenomic profiles of differentially abundant pathways between tracheal aspirates and fecal samples. Features with q < 0.1 were considered significant and thus retained. A positive difference between the proportions indicates a higher abundance in Fecal (orange), while a negative difference between the proportions indicates a higher abundance in Tracheal (blue). Tracheal, Tracheal aspirates samples; Fecal, Fecal samples.

Based on the MetaCyc database, the microbial metabolic pathways of all samples were predicted through PICRUSt. First, principal coordinates analysis (**Figure 5A**) was performed based on the overall results obtained from the PICRUSt functional prediction. It can be seen from the figure that the points representing fecal samples and the points representing tracheal aspirates samples are mixed to a certain extent, indicating that the microbial community structures of fecal samples (Feces) and tracheal aspirates samples (Tracheal) have overlapping parts, but there are also certain differences.

Furthermore, a heatmap (**Figure 5B**) reflecting the main functions predicted by the KEGG metabolic pathways was generated. These functions include: Caprolactam degradation, Phenylalanine metabolism, Styrene degradation, Cyanoamino acid metabolism, Tryptophan metabolism, Bacterial chemotaxis, Geraniol degradation, Fructose and mannose metabolism, Starch and sucrose metabolism. Bacterial chemotaxis and Geraniol degradation are the main components.

Pairwise comparisons were carried out using the prediction difference analysis of the KEGG metabolic pathways to compare the differences in bacterial functions among different samples. In the KEGG metabolic pathways, the classification, differences, and proportions of the KEGG metabolic pathways showing significant differences between groups are shown in **Figure 5C**. The comparison between the fecal group and the tracheal aspirates group showed that there were significant differences in 22 functional levels between the two groups. Among them, 13 were more abundant in the tracheal aspirates samples. Between the two groups, the functions of “Secondary bile acid biosynthesis”, “Phosphotransferase system (PTS)”, and “Bacterial chemotaxis” had the most significant differences. There were no significant differences in functions such as “metabolic Apoptosis” between the two groups.

### 3.6 Bacterial biomarkers in the control and patients with ALRTI

**Figure 6:**
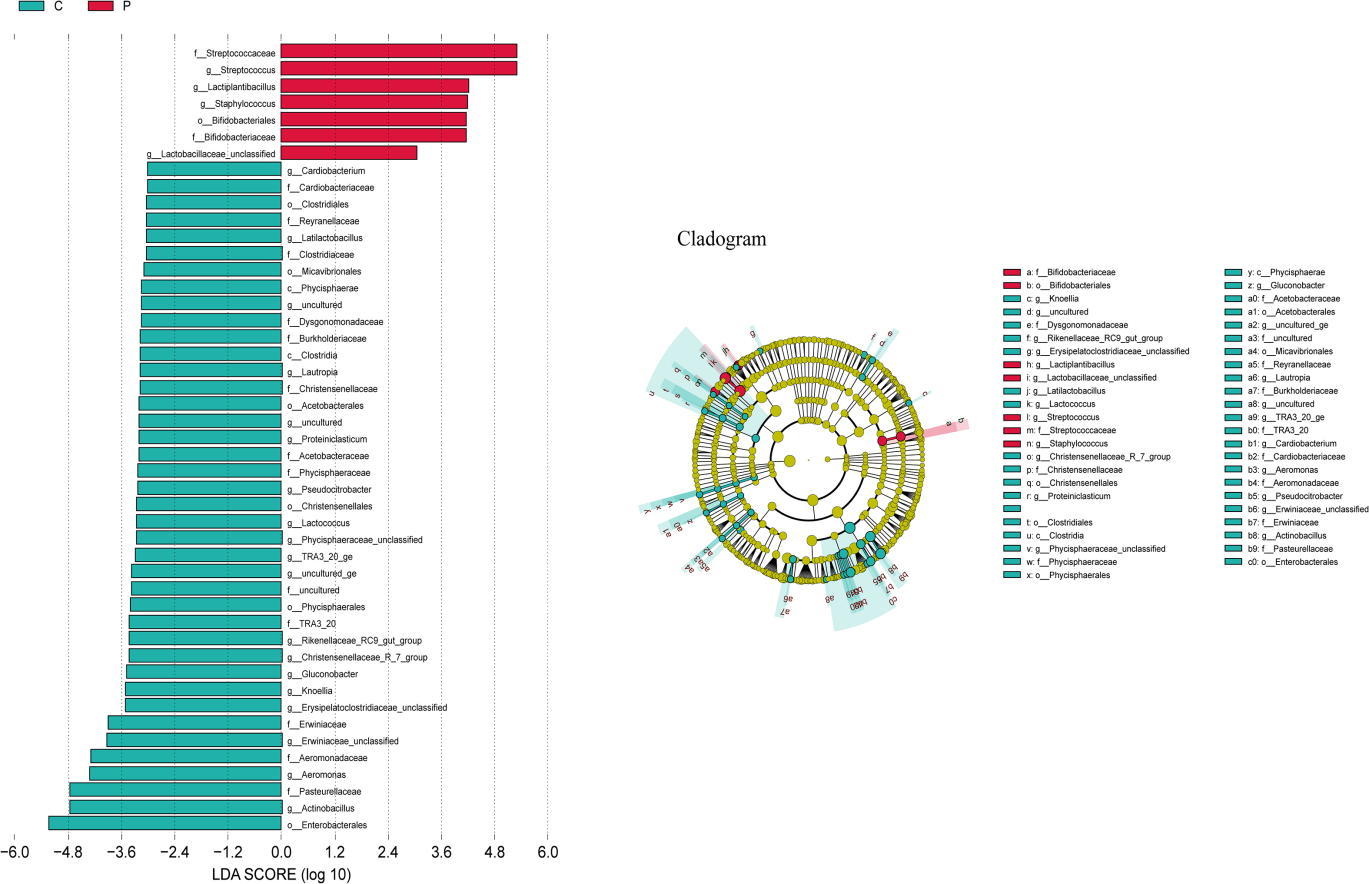
Bacterial biomarkers were identified by linear discriminant analysis effect size (LEfSe) algorithm. Differences in the relative abundance of microbial between the two groups; C, Control group; P, Patient.

The LEfSe algorithm was further utilized to analyze the bacterial community structures associated with the control group and ALRTI group, aiming to identify potential candidate bacteria as biomarkers related to respiratory diseases. As shown in **Figure 6**, a total of 23 different genera were identified. For ALRTI, there were 4 identified potential biomarkers, mainly including *Streptococcus*, *Lactiplantibacillus*, *Staphylococcus*, and *Lactobacillaceae*_unclassified. In the control group, a total of 19 potential biomarkers were identified, including genera such as *Actinobacillus*, *Aeromonas*, *Knoellia*, *Gluconobacter*, *Lactococcus*, *Pseudocitrobacter*, *Proteiniclasticum*, *Lautropia*, *Cardiobacterium*, and *Latilactobacillus*.

### 3.7 Bacterial composition and hierarchical clustering analysis of twin neonates

**Figure 7:**
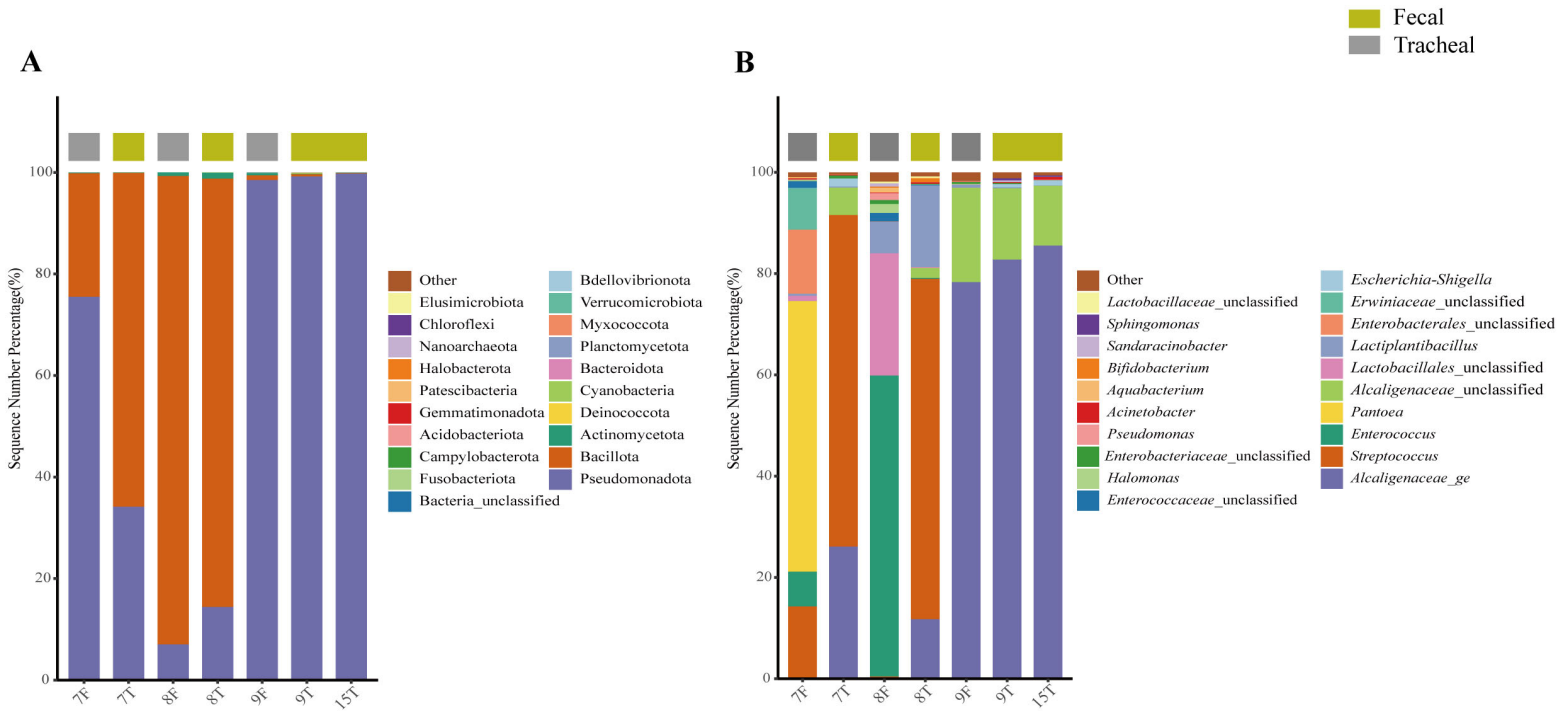
Composition of fecal and tracheal aspirates microbial structure in two pairs of twins; The bacterial community structure of seven samples from twin neonates was analyzed at the phylum level **(A)** and genus level **(B)**. T, Tracheal aspirates samples; F, Fecal samples.

The origin of the respiratory tract bacteria was studied 9–12 minutes after the birth of the twin neonates. The bacterial compositions in the airway secretion and fecal samples of two pairs of twin neonates were analyzed and compared. Numbers 7 and 8 were a pair, called the first pair of twins. Numbers 9 and 15 were a pair, called the second pair of twins; the fecal sample of number 15 was not collected. The results showed that the bacterial compositions of the twins were very similar. First, at the phylum level (**Figure 7A**), the bacterial compositions of the airway secretion samples of the two pairs of twin neonates were different, but the compositions of each pair of twins were similar. In addition, the fecal sample composition of each pair of twins was similar to that of its airway secretion sample. At the genus level (**Figure 7B**), *Enterococcus* was the same component in the fecal samples of the first pair of twins. Interestingly, the bacterial composition of the fecal samples of the first pair of twins was different from that of the airway secretion samples, but *Streptococcus* and *Lactobacillus* were the common components in the fecal and airway secretion samples of the first pair of twins respectively. The bacterial composition of the fecal sample of neonate 9 was the same as that of the airway secretion sample, mainly consisting of *Alcaligenaceae_*ge.

## 4 Discussion

ATIs are mainly caused by viral infections and coinfections with bacterial pathogens (Florin et al., 2017). Viral and bacterial infections have been shown to induce an immune response and microbiota imbalance (Brealey et al., 2015). The current study revealed that the feces and airway secretions microflora of diseased neonates were different from those of control neonates. Compared with the control neonates, the diversity of the bacterial community in the diseased neonates was lower, which may be due to the overgrowth of some particular taxa that tips the balance toward respiratory symptomatology, either by direct action as invasive pathogens or via indirect dysregulation of the local immunological milieu (Teo et al., 2015). In addition, it was found that the similarity between the feces and airway secretion microbiota in diseased neonates can be explained by a robust intrusion of pathogens through the mouth and transmission from the mouth to the stomach and lung.

The current study provided information regarding the bacterial community of neonatal feces and airway secretion samples. It was found that the composition of the bacterial community in every individual was different; this also demonstrated the heterogeneity between subjects (Brusselaers et al., 2013). *Enterococcus* was the most abundant genus in the feces samples of diseased neonates, It often induces antibiotic resistance due to the abuse of antibiotics and may cause fatal nosocomial infections in humans (Wu et al., 2013; Hendrickx et al., 2015). *Achromobacter* was dominant in the feces and airway secretion samples of diseased neonates, it is a known multidrug-resistant nosocomial and cystic fibrosis pathogens, which may also cause community-acquired respiratory disease in subjects without cystic fibrosis (Swenson and Sadikot, 2015). All members of the *Achromobacter* genus possess motile peritrichous flagella that contribute to potential biofilm formation, a necessary component for airway colonization, infection, and resistance of the microbe to antibiotic agents, and may assist with host cell invasion (Jakobsen et al., 2013). Similar to other gram-negative pathogens, *Achromobacter*-derived LPS induces key inflammatory cytokines, such as IL-6, IL-8, and TNF (Hutchison et al., 2000). Another potential pathogenic bacterium identified was *Streptococcus*, which has been shown to be dominant in the oropharynx microbiota profile of both hospitalized and healthy infants (Hu et al., 2017); several members of *Streptococcus* spp. could induce inflammation in the epithelial mucus (Yoo et al., 2010). Numerous reports have described the oropharynx microbiota in healthy infants, and it was primarily dominated by *Streptococcus, Rothia, Gemella*, and *Neisseria* (Stearns et al., 2015). The current study has shown that microbial carriage in the airway secretion samples of control neonates was identical to that described in these reports. Several reports have identified four microbiota profiles in the nasopharynx of infants hospitalized for bronchiolitis, and the *Haemophilus*-dominated profile was associated with the highest clinical severity (Biesbroek et al., 2014; Hasegawa et al., 2016a, Hasegawa et al., 2016b). This study established an *Achromobacter*-dominant airway secretion microbiota profile in diseased neonates, which could be attributed to the differing microbiota components of the nasopharynx and tracheal aspirate (Charlson et al., 2011; Bassis et al., 2015). Therefore, the airway secretion microbiota has promising potential in bronchiolitis prognosis, therapy optimization, and evaluation of recovery.

Growing evidence suggests that the source of early microorganisms may influence neonate immunity and thus impose important consequences for disease susceptibility (Vissing et al., 2013). This study revealed that multiple bacterial taxa can be identified in the respiratory secretions of intubated neonates, even at the time of birth. This was interesting even though all neonates in this study were delivered by cesarean section, most membranes were intact and not in labor. This finding differed from the data reported by (Mourani et al., 2011), in which there was an insufficient quantity of bacterial DNA identified via sequencing prior to 72 hours in most of the specimens. The results of this study were consistent with findings by DiGiulio et al., who suggested associations between the presence of organisms or bacterial DNA in amniotic fluid and clinical outcomes (DiGiulio et al., 2008). Furthermore, the current results indicated that twins had a more similar bacterial composition and that risk factors were the main factors affecting different patients and the main species present in the samples. This suggested that these babies may be more susceptible to the influence of early microbial exposure prior to delivery. Of course, the effects of genetic and/or family factors on the lungs and intestines of twins were not excluded. An association between caesarean section and respiratory symptoms in early life has been reported (Magnus et al., 2011), and the microbial sources of infants born by cesarean section were different from those of infants born naturally (Dominguez-Bello et al., 2010). Further studies are needed to pursue the link between the role of the human microbiome, immune maturation, and disease development.

In this study, preliminary differences in microbial structure were observed between the preterm and non-preterm groups on Axis 1, but these differences were not statistically significant (P = 0.352), and the explanatory power of the grouping was low (R² = 0.035). This indicates that the “N/P” factor is not the main driver of community changes. This finding is inconsistent with the results of (Ardissone et al., 2014), who suggested that preterm birth has a significant impact on the fetal fecal microbiota. However, the overlap observed in this study implies that other unmeasured environmental variables or random processes may have a stronger influence on community formation. Future studies should include more covariates (such as maternal lactation diet and feeding methods) to clarify the key determinants of microbiome structure.

The current results demonstrated features of lower respiratory tract bacterial community structure (i.e., diminished alpha diversity and a single, dominant taxon) that may prove useful in the diagnosis of lower respiratory tract illnesses (LRTIs) in intubated subjects, a group in which an LRTIs diagnosis was particularly challenging (Klompas et al., 2008). Sequence-based analysis of the respiratory microbiome had several other potential advantages in critically ill individuals. The parturients received broad-spectrum antibiotics during the cesarean section. Antibiotics may turn cultures negative, but since sequence analysis did not depend on bacterial viability, this approach may still identify dominant organisms. A routine culture had indicated which subjects were clinically suspected of having LRTIs but had multiple negative clinical cultures obtained from the lower respiratory tract; thus, microbiota analysis can be complementary to clinical detection of respiratory pathogens. In contrast, 16S rRNA gene sequence analysis captured all bacteria present, and relative abundance measures had the potential to reveal outgrowth and dominance by any taxon; these findings would have allowed earlier interventions. In addition, detailed early knowledge of dominant organisms would have allowed use of more precisely targeted antibiotic therapies, thereby minimizing activity against the normal microbiota and the development of antibiotic resistance;

However, there are some limitations to this study. First, in this study, samples in the respiratory tract infection group were collected from airway secretions, while samples in the control group were oral swabs. We acknowledge that there are significant differences in anatomical structure and microenvironment between the upper and lower respiratory tracts (Marsh et al., 2016) (such as oxygen levels and mucosal immune status). These differences may lead to inherent variations in the microbial community composition between the two groups of samples, which could partially interfere with the analysis of the association between infection status and microbial communities. The discrepancy in sample sources constitutes a limitation of this study. The use of oral swabs as samples from control neonates was primarily due to ethical constraints and operational difficulties in obtaining lower respiratory tract samples from control neonates in clinical settings. Future studies could further optimize the experimental design, such as using matched upper respiratory tract samples (e.g., nasopharyngeal swabs) as controls, or including lower respiratory tract samples from control neonates when conditions permit. This would help reduce the impact of differences in sample sources on the interpretation of results. Secondly, the small sample size of this study may also lead to limitations in the conclusions (e.g., it may not be fully generalizable to all neonates with ALRTI). The insufficient sample size is due to the difficulty in obtaining samples from neonates in the control group; moreover, to avoid the impact of environmental factors during sampling at different time periods, it was necessary to ensure that samples from control neonates and those from ALRTI patients were collected within the same time period. Therefore, only two non-pneumonia control samples were obtained during the sampling period in our department. In future studies, we will make every effort to collect more control samples. Finally, clinical symptoms and host responses, including immune cytokines, should be fully considered to ensure the accuracy of microbiome analysis. For further studies, larger sample collections are needed. In addition, the influence of sample type needs to be fully considered. In addition, molecular analysis offers valuable information not available through traditional respiratory tract cultures. However, it would best serve as a complement to cultures, as determination of antibiotic sensitivity currently still requires culture-based analysis.

This study explored the bacterial composition of feces and airway secretion samples in Chinese neonates with ALRTI and provided additional reference data for associated studies. More importantly, the source of pathogens was analyzed by comparing the bacterial composition of airway secretion and feces in two pairs of neonates twins. The pathogenesis of ALRTI is complex, and these findings suggest that the initial exposure to bacterial products may not be central to lung injury but rather affect the immune response and microbiome succession. High-throughput sequencing technology has allowed us to identify a possible clinically relevant microbiome in neonates, but further studies are needed to improve our understanding of the bacteria–host interactions and to enable interventions in the sequence of lung injury.

## Data Availability

The datasets presented in this study can be found in online repositories. The names of the repository/repositories and accession number(s) can be found below: https://www.ncbi.nlm.nih.gov/, SRP219865.
